# Validation of prototype virus inactivation from seven virus families of pandemic potential with a novel low-cost, field-deployable RNA extraction and storage method

**DOI:** 10.1016/j.jviromet.2025.115292

**Published:** 2025-10-27

**Authors:** Michelle L. Rock, Jessica B. Huskey, Sarah Hernandez, Divya P. Shinde, Thomas H. Oguin, Sara Ping, Arabella Lewis, Jesse J. Waggoner, M. Anthony Moody, Gregory D. Sempowski, Brook E. Heaton

**Affiliations:** a Duke Human Vaccine Institute, Duke University School of Medicine, Durham, NC, United States; b Emory University School of Medicine, Atlanta, GA, United States; c RTI International, Research Triangle Park, NC, United States

**Keywords:** RNA extraction, Virus inactivation, Field surveillance, Emerging infectious diseases, Pandemic preparedness

## Abstract

The Centers for Research in Emerging Infectious Diseases (CREID) was established to enhance pandemic preparedness by studying emerging/reemerging pathogens, especially in resource-limited regions. To overcome infrastructure challenges, a low-cost, field-deployable method for extracting total nucleic acids is essential, eliminating reliance on expensive equipment, power, and cold chain systems used in traditional extraction techniques. To address this challenge, we developed an RNA extraction and storage method (RNAES) that meets these criteria. Herein, we report RNAES inactivation efficacy against nine prototype viruses (Middle Eastern respiratory syndrome coronavirus, Japanese encephalitis virus, West Nile virus, Hantaan virus, measles virus, Heartland virus, enterovirus A71, chikungunya virus, and Western equine encephalitis virus) representing seven pandemic potential virus families. We compare the RNAES method to the Qiagen QIAamp kit across various viral loads and field sample types. The presence of infectious virus in RNA samples was quantified using plaque assays. Successful inactivation of viruses was demonstrated for six enveloped virus families spiked into matrices routinely collected at field sites. The seventh family tested (*Picornaviridae*) was not completely inactivated, likely due to non-enveloped viruses being differentially susceptible to the lysis chemistry of the RNAES kit. The commercial comparator inactivated all viruses tested. Specialized biosafety facilities, specific detailed permits, and comprehensive logistics are required to ensure safety when handling and shipping potentially infectious samples. Inactivating pathogens at the point of collection reduces risks and simplifies sample transfer for critical outbreak research. Confidently ensuring that an isolated nucleic acid sample is non-infectious using RNAES will enable safer, and more efficient downstream analysis.

## 1. Introduction

Foundational goals of the National Institute of Allergy and Infectious Diseases (NIAID) mission and alignment with the NIAID Pandemic Preparedness Plan include expanding the breadth and depth of knowledge in infectious diseases and developing flexible domestic and international research capacities to respond appropriately to global emerging and reemerging infectious disease (re/EID) threats (National Institute of Allergy and Infectious Diseases NIAID, 2021a, 2021b). Recent emergences of viral, zoonotic-origin infectious pathogens, such as SARS-CoV-2 (Muralidar et al., 2020), oropouche (Scachetti et al., 2025), and yellow fever virus (Faria et al., 2018), have demonstrated their ability to significantly impact human health and quality of life. Past outbreaks revealed inefficiencies in communication, coordination, and collaboration between unlinked investigators or research networks and in-country public health response entities, which made it difficult for NIAID to initiate collaborative outbreak-related research activities quickly and efficiently.

In 2020, NIAID funded the Centers for Research in Emerging Infectious Diseases (CREID) Network to address these challenges and to enhance global pandemic research preparedness and response with the development of an adaptable, scalable, and sustainable infrastructure for global infectious disease research before, during, and after outbreaks. CREID is composed of nine Research Centers with more than 115 sites strategically located in infectious disease hot spots in over 30 countries. The global network with more than 350 investigators and staff is coordinated by a central Coordinating Center. CREID multidisciplinary teams conduct surveillance, transmission, pathogenesis, and host immune response research to achieve an overall goal to proactively identify and respond to global outbreaks and gain mechanistic insight to facilitate development of diagnostics, countermeasures, and vaccines for future outbreaks. The CREID research network is uniquely positioned to rapidly coordinate and facilitate in-country surveillance and outbreak adjacent research on re/EID virus families of pandemic potential in alignment with the World Health Organization Research and Development Blueprint (Pathogens Prioritization: A scientific framework for epidemic and pandemic research preparedness) (World Health Organization, 2024).

CREID and other re/EID investigators must work in areas of the world where pathogens are emerging or spilling over. These areas of the world are often underdeveloped and offer limited resources, infrastructure, and specialty equipment. To overcome these challenges, it is imperative to have a low-cost, field-deployable method to extract viral nucleic acids, that is uncoupled from traditional methods that may require expensive equipment, power, and sophisticated cold chain systems. This method should allow field operatives to extract genomic material from a variety of sources (e.g., animal/human hosts, vectors, and the environment) and then safely send that material to local or distant collaborating laboratories for analysis, such as viral genome sequencing, to rapidly identify present viruses.

Unfortunately, safe handling and transfer of potentially infectious specimens across local, national, and international borders can require complex and specialized facilities and capabilities. Furthermore, transfer of known infectious materials requires substantial import/export permitting and expensive shipping logistics. To mitigate these barriers to efficient outbreak research, validated inactivation of infectious pathogens in the sample at the point of collection can be employed to minimize risk and streamline the ability to transfer the extracted and stabilized material. Knowing with confidence that the extracted nucleic acid sample no longer contains infectious virus can allow analysis to be done more quickly and efficiently.

To address this challenge, investigators in the American and Asian Centers for Arboviral Research and Enhanced Surveillance, one of the nine CREID Network research centers, developed a field-deployable RNA extraction and storage method (RNAES) (Hernandez et al., 2022; Hernandez et al., 2025). The method was designed to use off-the-shelf items readily available to most CREID or other re/EID research and field-collection sites. Critical to the utility of this RNA extraction and storage method for CREID, and the greater emerging infectious diseases community, is validating that the procedure can inactivate a range of Network-relevant family-level prototype viruses.

Herein, we report efficacy of a novel RNAES method (Baer et al., 2024; Hernandez et al., 2022, 2023), and a commercial comparator (Qiagen QIAamp), on inactivating nine prototype or surrogate viruses from seven virus families. The selected viruses represent a cross-section of pathogens that pose ongoing or potential threats to global health. Many have caused significant outbreaks or epidemics in recent decades. Collectively, these viruses highlight the breadth of challenges facing public health: zoonotic spillover, vector-borne transmission, and person-to-person spread. By including viruses with different structures, transmission routes, disease severities, and geographic distributions, the selected panel ensures that the evaluation of the extraction method reflects real-world scenarios in which diverse viral threats could emerge (Table 1). Inactivation testing with both nucleic acid extraction methods was performed across a broad range of potential viral loads in mock field sample types (i.e., host blood, host serum, viral transport media (VTM), vector pool, and environment) (Table 2).

## 2. Materials and methods

All live virus laboratory work was performed in the Duke Regional Biocontainment Laboratory under BSL-2 or BSL-3 containment with a comprehensive biosafety and biosecurity oversight program.

### 2.1. Viruses and cell lines

Nine pandemic potential prototype pathogens representing seven virus families were selected to assess viral inactivation efficiency by a novel nucleic acid extraction method and commercial kit comparator. Seed stocks of each virus were acquired from the Biodefense and Emerging Infections Research Resources Repository (BEI): Middle Eastern respiratory syndrome coronavirus (MERS-CoV; NR-48813), Japanese encephalitis virus (JEV; NR-2331), West Nile virus (WNV; NR-677), Hantaan virus (HTNV; NR-9370), measles virus (MeV; NR-3847), Heartland virus (HRTV; NR-49771), enterovirus A71 (EV A71; NR-52000), chikungunya virus (CHIKV; NR-13220), and Western equine encephalitis virus (WEEV; NR-41569).

Vero E6 cells (ATCC CRL-1586) were maintained in complete growth medium (minimum essential medium [MEM; Gibco 11095–080] + 10 % heat-inactivated fetal bovine serum [FBS; Gemini 100–106] + 1 % Penicillin Streptomycin [P/S; Gibco 15140–122] + 1 % MEM Non-Essential Amino Acids [NEAA; Gibco 11140–050] + 1 % sodium pyruvate [Gibco 11360–070]) in T-175 cell culture flasks (Corning 431080) at 37°C, 5 % CO_2_ in humidified incubators. As needed, working stocks of each virus were generated using Vero E6 cells. Briefly, stock virus was diluted in virus diluent (VD1; MEM + 2 % FBS + 1 % P/S + 1 % NEAA + 1 % sodium pyruvate) to the desired multiplicity of infection (MOI) (Table 3) in 5 mL of VD and added to cells of a confluent T-175 flask of Vero E6 cells. Infected flasks were then incubated for 1 h at 37°C, 5 % CO_2_ and tilted every 10–15 min. After the initial infection incubation, an additional 20–25 mL of VD was added, and the flask was returned to the incubator. Cytopathic effect (CPE) was observed regularly by light microscopy, and supernatant was collected when CPE exceeded 80 % (Table 3). Harvested supernatant was clarified by centrifugation (5 min at 500 x g) and vacuum filtered through a 0.22 μm polyethersulfone (PES) membrane (Corning, 431153). The exception to this protocol was Hantaan virus, which does not produce CPE in cell culture (Ma et al., 2017) and was instead incubated for 12 days with a VD change on day 7 before the supernatant was harvested. These virus stocks were aliquoted and stored at −80°C, and viral titers were quantified by plaque assay.

### 2.2. Plaque assays

Optimized, virus-specific plaque assays were used to measure the viral titer of each stock produced. The appropriate overlay viscosity and incubation time for each virus are listed in Table 3. Briefly, Vero E6 cells were seeded in flat, 12-well tissue culture plates 1 day prior to the assay. Virus was serially diluted 10-fold in the appropriate VD depending on virus (Table 3) (VD1: see above; VD2: MEM + 0.12 % bovine serum albumin [BSA; Gibco 15260–037] + 1 % P/S + 1 % NEAA + 1 % sodium pyruvate + 1 % HEPES buffer solution [Gibco 15630–080]), and viral dilutions were added to duplicate wells of Vero E6 cells. Plates were incubated for 1 h at 37°C and rocked every 10–15 min, then overlaid with a viscous solution of methylcellulose (MC) (Sigma M0512) diluted in 2X MEM (20 % 10X MEM [Gibco 11430–030] + 4 % FBS + 2 % P/S + 2 % NEAA + 2 % sodium pyruvate + 8 % sodium bicarbonate [Gibco 25080–094] + 2 % L-glutamine + 60 % cell culture grade water) according to virus (Table 3). After incubating, each plate was fully sub-merged in 10 % neutral buffered formalin for at least 30 min. For all viruses, except HTNV, plates were then stained with 0.1 % crystal violet in water for at least 5 min. Excess stain was removed by washing with tap water. After drying, plaques in the cell monolayer were counted with the aid of a white-light transilluminator, and duplicate viral titers were averaged to determine the plaque forming unit (PFU) per milliliter titer for each working stock. Plaque assay plates for HTNV were immunostained using an anti-Hantavirus primary antibody (Medix Biochemica V4956) at a 1:2000 dilution and a horseradish peroxidase (HRP) conjugated goat anti-mouse IgG secondary antibody (SeraCare 95058–740) at a 1:2000 dilution, then developed with True Blue HRP substrate (SeraCare 95059–168). Foci in the cell monolayer were counted and duplicate viral titers were averaged to determine the foci forming unit (FFU) per milliliter titer for the HTNV stock.

Optimized plaque assays for all prototype viruses were also used to evaluate the eluates from each RNA extraction for infectious virus. Both RNA extraction methods described below produced 100 μL of sample eluate. After removal of the culture medium from cell plates, the entire volume of sample eluate was plated into one well of an assay plate with an additional 150 μL of appropriate VD (VD1 or VD2) to prevent the cell monolayer from drying out. Positive controls consisted of virus diluted in the same manner as the test samples (800 μL high, medium, or low virus added to 1200 μL VD). These samples were then diluted 1:10 serially to ensure countable results and 25 μl was used to infect each plaque assay. Negative control plates included wells of 200 μL of VD or 100 μL of TE buffer (10 mM Tris, 1 mM EDTA, pH 8.0) supplemented with 100 μL of VD added to each well. All plaque assay plates were incubated at 37°C, 5 % CO_2_ in a humidified incubator for 1 h, with rocking every 10–15 min followed by the addition of overlay and incubated as detailed in Table 3. Plates were then fixed with 10 % neutral-buffered formalin for at least 30 min and stained with 0.1 % crystal violet or immunostained (Table 3). The number of PFU or FFU in each well was determined by counting the number of plaques/foci and adjusting for applicable dilution factors. Standard error of the mean (SEM) was calculated in Microsoft excel by dividing the standard deviation of the sample by the square root of the sample size. Wells exhibiting disruption of the cell monolayer attributable to technical issues were excluded from the analysis.

### 2.3. Mock surveillance specimen generation

For each prototype virus, bulk lots of mock surveillance specimens in five matrices (Table 2) at three representative viral titer levels (i.e., high, medium, low) and a negative were prepared depending on maximum stock titer available. The negative is a matrix-only control. Each inactivation test sample was 25 μL, according to the maximum sample loading volume for the RNAES method. The 25 μL sample consisted of 10 μL of stock virus and 15 μL of one of the following five matrices: human whole blood (Innovative Research IWB1K2E10ML), human serum (Sigma Aldrich H5667–mL), Viral Transport Medium (HBSS [Gibco 24020–117] + 2 % FBS + 1 % P/S + 5 % artificial saliva [Pickering 1700–0316]), homogenized mosquitos (15–20 mosquitos/mL PBS) (*Aedes aegypti* BEI MRA-735B and *Anopheles albimanus* BEI MRA-133B) homogenized in Bertin Corp Hard Tissue Homogenizing tubes (Fisher 50–154–2931), and pond water (locally acquired). These volumes were scaled up so that 800 μL of virus (neat, diluted 1:2, or diluted 1:4 for the high, medium, and low viral load respectively) was added to 1200 μL of matrix. The negative control for each matrix was prepared in the same fashion by adding 800 μL of VD to 1200 μL of each matrix. The positive control viruses for plaque assay were also prepared in the same way by adding 800 μL of each virus amount (i.e., high, medium, or low) to 1200 μL VD. This preparation allows 25 μL of the control to be added to the plate to represent the same amount of virus that was used in the inactivation protocols. When all samples were prepared, 200 μL aliquots were made and stored at −80°C until use in the inactivation testing studies.

### 2.4. RNA extraction

RNAES Method: Three replicates of each prototype virus-spiked mock sample (i.e., high, medium, low, and negative for each matrix) were processed using the RNAES protocol as previously described (Hernandez et al., 2022). Prior to beginning the RNA extraction, each RNAES cartridge was prepared with a GF/D membrane (Sigma WHA1823042) and a blotting pad (VWR 28298–014). For extraction, one lyophilized maltodextrin lysis sphere (EVIK Diagnostics) containing potassium chloride with sucrose, proteinase K, and carrier RNA was added to each nuclease-free sample tube. Test sample was added (25 μL) and mixed by flicking the tube. The lysis mixture was incubated at room temperature for at least 10 min prior to adding 250 μL of arginine binding buffer (40 % 500 mM L-Arginine HCl; 250 mM KCl; 250 mM MgCl_2_, 60 % 200 proof ethanol). The contents of the tube were then transferred dropwise to the RNAES cartridge membrane. Once the sample had gone through the membrane, 100 μL of 0.1 M glycine-HCl buffer was added to the membrane and incubated for 10 min at room temperature to ensure that the membranes were free of glycine wash buffer, which is toxic to the cells used in the plaque assay. After the incubation, the membranes were moved to 1.5 mL microcentrifuge tubes containing 100 μL of TE buffer (10 mM Tris, 1 mM EDTA, pH 8.0) and incubated at room temperature for at least 1 min for elution. After incubating, the membranes were discarded and the microcentrifuge tubes stored at 4°C until samples were run in a plaque assay.

Qiagen QIAamp Method: Three replicates of each prototype virus-spiked mock sample (i.e., high, medium, low, negative for each matrix) were also processed using the Qiagen QIAamp 96 Viral RNA kit (52962) according to manufacturer instructions. Briefly, carrier RNA was added to AVL Lysis Buffer at 10 μg/mL. The complete lysis buffer was aliquoted 100 μL per tube and 25 μL of test specimen was added, maintaining a critical one-part sample to four-parts lysis buffer ratio. The reaction was incubated at room temperature for 10 min before 100 μL of 200 proof ethanol was added. The entire sample was transferred to a QIAamp 96 plate, which was then covered with airpore tape and attached to a collection plate. Plates were centrifuged at 2272 × g for 5 min and the flow-through discarded. These steps were repeated after each wash, the first with 200 μL AW1 per well and the second with 200 μL of AW2 per well. The plate was then centrifuged for a drying step to ensure that no wash buffer remained in the membrane. For the elution step, the QIAamp 96 plate was attached to a fresh collection plate and 100 μL of nuclease-free water was added to each well, incubated for 1 min at room temperature, and then centrifuged. The collection block with the eluates was sealed with an aluminum plate cover and stored at 4°C until samples were run in a plaque assay.

### 2.5. Quantitative reverse transcriptase polymerase chain reaction (RT-qPCR)

High concentration samples of all prototype viruses from all matrices were processed and then stored on dried RNAES membranes in tubes with desiccant for RT-qPCR verification of extracted viral RNA. To prepare samples for RT-qPCR, membranes were removed from storage tubes and incubated for 60 s in 100 μL of 1X TE buffer in clean 1.5 mL tubes. Membranes were discarded, and 5 μL of the resulting eluates were used in 20 μL RT-qPCR reactions of the Luna Universal One-Step RT-qPCR Kit (New England Biolabs E3005) on a Rotor-Gene Q instrument (Qiagen). Detection by RT-qPCR was defined by an exponential curve that crossed the amplification threshold prior to cycle 40, consistent with previously published criteria for detection (Waggoner et al., 2016). Cycle thresholds were set manually to cross amplification curves at the start of the logarithmic phase of amplification. Primers and probes were used as previously described (Brault et al., 2015; Broadhurst et al., 2021; Dierssen et al., 2008; Fajfr et al., 2014; Gomez et al., 2015; Savage et al., 2013, 2016; Waggoner et al., 2016).

## 3. Results

**Fig. 1.**
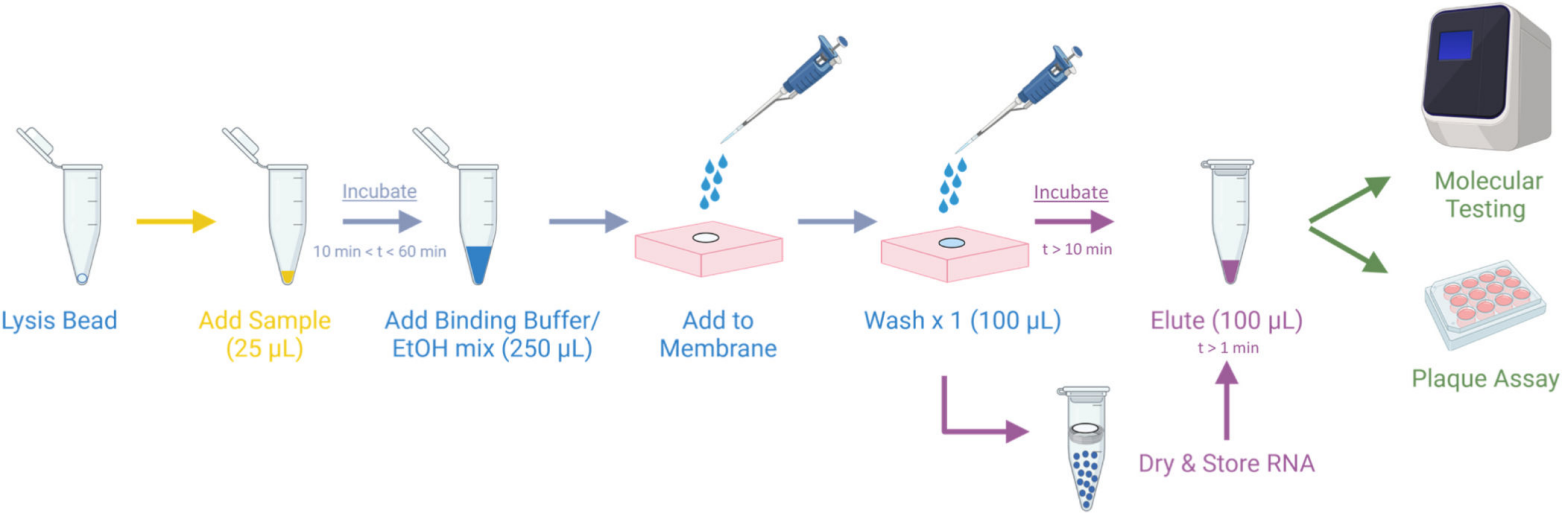
Schematic of RNAES extraction method (Created with BioRender.com). Sample was added to a lysis bead and incubated at least 10 min. A binding buffer supplemented with ethanol (EtOH) was added to the sample and the sample was moved to the membrane. One glycine wash was added to the membrane before the membrane can either be eluted or stored in a tube with desiccant beads and dried for later elution. The resulting eluate can be used in a plaque assay or for molecular testing.

Mock field specimens (Table 2) were prepared with high, medium, and low viral loads with nine prototype viruses from seven high-consequence virus families (Table 1). Specimens were processed for RNA extraction using both the CREID-developed RNAES method (Fig. 1) and a commonly used commercial comparator kit (Qiagen QIAamp). Resulting eluates were then tested by plaque assay to determine whether infectious virus transferred with the eluted RNA. Detailed below are the results of this robust inactivation efficacy testing across family-level prototype pathogens. Each assay included three controls: (i) a negative extraction control (matrix only without virus that was then processed through the RNAES or QIAamp kit and tested by plaque assay); (ii) a positive plaque-assay control using diluted virus, used without extraction to confirm plaque assay conditions; and (iii) a negative plaque-assay control using virus diluent (VD) only, to confirm that plaque assay conditions were appropriate.

### 3.1. Coronaviridae – MERS-CoV

**Fig. 2.**
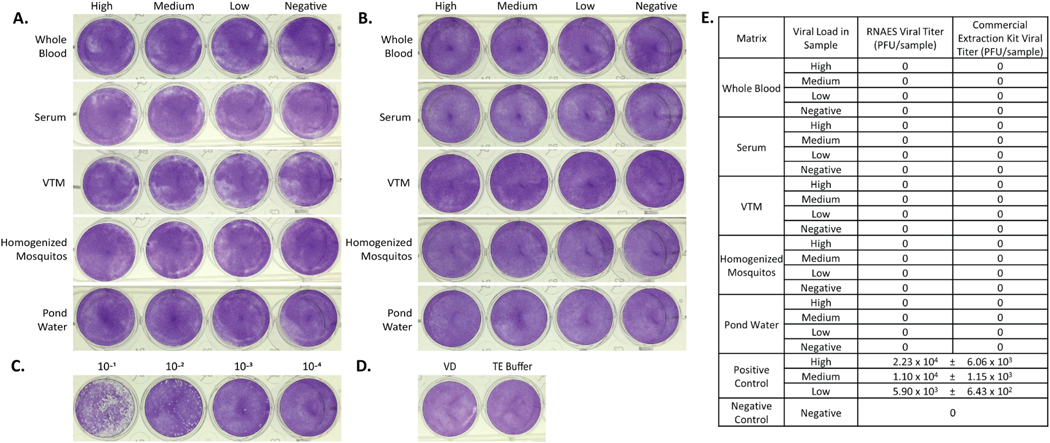
MERS-CoV is inactivated by both RNAES and Qiagen QIAamp extraction kits. MERS-CoV was spiked into five matrices with high, medium, low, and negative viral loads, followed by inactivation by two RNA extraction methods. Inactivation of eluates from the (A) RNAES and (B) Qiagen RNA extraction kits was tested using a crystal violet stained plaque assay (n = 9). (C) A positive control (n = 3) was included, the high titer positive is pictured, as well as (D) a VD negative control (n = 6) and a TE buffer negative control (n = 6). (E) Summary of plaque assay results; titers are reported as mean ± SEM.

Five virus-spiked matrices (human whole blood, human serum, VTM, homogenized mosquitos, and pond water) were prepared at high, medium, low, and negative concentrations of MERS-CoV virus per 25 mL of sample. VTM was employed as representative sample matrix for respiratory swab samples, which are typically placed into VTM/UTM (Universal Transport Media) or saline prior to processing. This also serves as a general sample matrix used in virology laboratories. For each spiked test specimen, RNA was extracted from three replicate samples, and the experiment was repeated three times for a total of nine replicates per condition using both the RNAES method and Qiagen commercial comparator. The entire elution volume from each RNA extraction was tested in plaque assay format to assess viral inactivation. No plaques were observed in any of the test conditions of eluates from RNAES (Fig. 2A). Similar results were seen with the commercial extraction kit (Fig. 2B). To serve as a positive plaque assay control, MERS-CoV diluted in VD using the same dilution factor as the virus spiked matrices, was plaqued and served as a positive control for detection and enumeration of virus. Plaque assay results of the high concentration of positive control virus, calculated to be 2.23 × 10^4^ PFU per sample, are shown in Fig. 2C. Negative controls for each plaque assay consisted of either VD or TE Buffer (the buffer RNA samples were eluted in) and were also included in the plaque assay (Fig. 2D). A summary of all plaque assay results for MERS-CoV is shown in Fig. 2E. The field-deployable RNAES and commercial comparator methods were both sufficient to inactivate up to 2.23 × 10^4^ PFU of MERS-CoV virus in all tested specimen matrices.

### 3.2. Flaviviridae – JEV and WNV

**Fig. 3.**
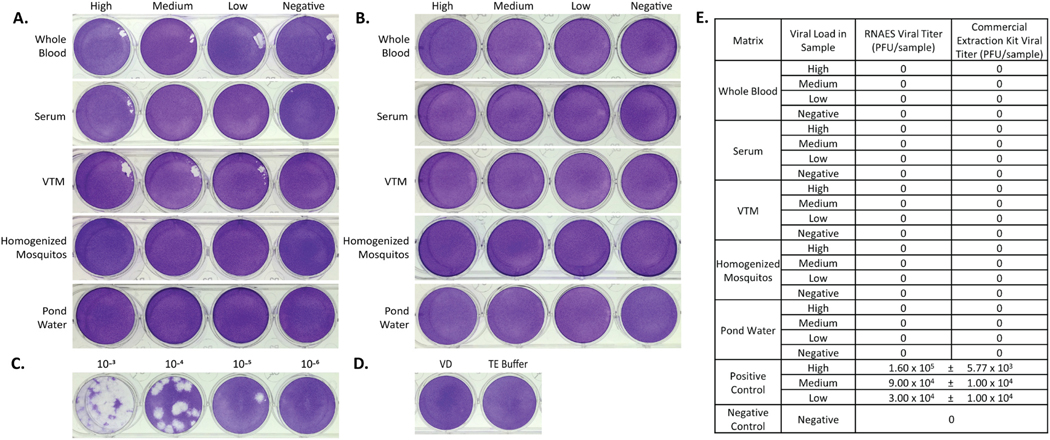
JEV is inactivated by both RNAES and Qiagen QIAamp extraction kits. JEV was spiked into five matrices with high, medium, low, and negative viral loads, followed by inactivation by two RNA extraction methods. Inactivation of eluates from the (A) RNAES kit from whole blood, VTM, homogenized mosquito, and pond water sample (n = 9) and serum samples spiked with high viral load (n = 8), medium viral load (n = 6) and low and negative viral loads (n = 9) was tested using a crystal violet stained plaque assay. Inactivation of eluates from the (B) Qiagen RNA extraction kit was also tested using a crystal violet stained plaque assay (n = 9). (C) A positive control (n = 3) was included and a representative high titer positive is depicted, as well as (D) a VD negative control (n = 6) and a TE buffer negative control (n = 6). (E) Summary of plaque assay results; titers are presented as mean ± SEM.

**Fig. 4.**
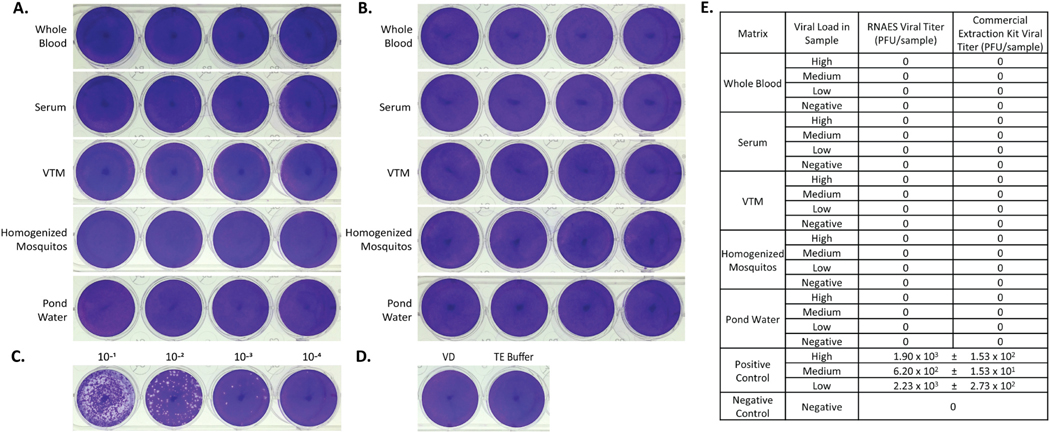
WNV is inactivated by both RNAES and Qiagen QIAamp extraction kits. WNV was spiked into five matrices with high, medium, low, and negative viral loads, followed by inactivation by two RNA extraction methods. Inactivation of eluates from the (A) RNAES and (B) Qiagen RNA extraction kits was tested using a crystal violet stained plaque assay (n = 9). (C) A positive control (n = 3) was included, depicted is an image of the high titer positive, as well as (D) a VD negative control (n = 6) and a TE buffer negative control (n = 3). (E) Summary of plaque assay results; values are reported as mean ± SEM.

Two prototype members of *Flaviviridae*, JEV and WNV, were used as inputs in inactivation studies using the RNAES platform and Qiagen commercial comparator. Following RNA extraction using the RNAES kit, no plaques were present when the eluates were tested in a plaque assay for JEV (Fig. 3A). Similarly, the eluates from the commercial comparator had no plaques (Fig. 3B). The high positive control was calculated to be 1.60 × 10^5^ PFU per sample (Fig. 3C) and the negative controls did not have plaques (Fig. 3D). A summary of the JEV plaque assay data can be found in Fig. 3E. The eluates from the WNV samples from RNAES (Fig. 4A) and commercial comparator (Fig. 4B) did not have plaques. The high positive control for WNV plaqued at 1.90 × 10^3^ PFU per sample (Fig. 4C). Additionally, no plaques were seen in the negative controls (Fig. 4D). A summary of all the plaque assay data is shown in Fig. 4E. These results indicate that the RNAES method and commercial comparator methods can inactivate at least 1.60 × 10^5^ PFU of JEV and 1.90 × 10^3^ PFU of WNV in the five tested matrices.

### 3.3. Hantaviridae – HNTV

**Fig. 5.**
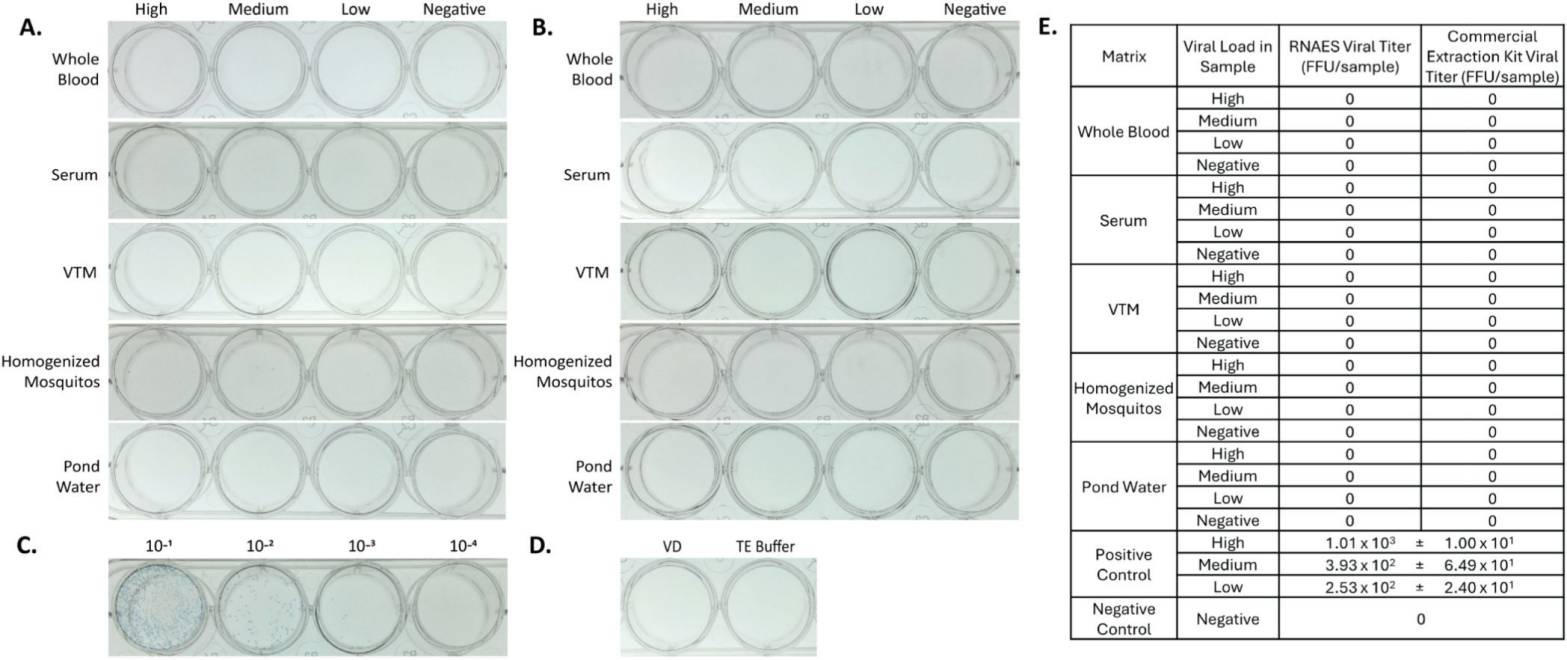
HTNV is inactivated by both RNAES and Qiagen QIAamp extraction kits. HTNV was spiked into five matrices with high, medium, low, and negative viral loads, followed by inactivation by two RNA extraction methods. Eluates from the (A) RNAES and (B) Qiagen RNA extraction kits were tested using an immunostained focus-forming assay (n = 9). (C) A high titer positive control (n = 3) was included, as well as (D) a VD negative control (n = 6) and a TE buffer negative control (n = 6). (E) Summary of assay results; results are reported as mean ± SEM.

Hantaan virus was used as a prototype member of the *Hantaviridae* family. RNA was extracted from high, medium, low, and negative viral load samples using RNAES and a commercial comparator. The resulting eluates from RNAES (Fig. 5A) and the commercial comparator (Fig. 5B) did not form foci. The high viral load positive control (Fig. 5C) had a titer of 1.01 × 10^3^ FFU per sample volume and the negative controls (Fig. 5D) did not have foci. A table summarizing all focus-forming assay results can be found in Fig. 5E. These results indicate that the RNAES method and commercial comparator methods can inactivate at least 1.01 × 10^3^ FFU of HNTV in the five tested matrices.

### 3.4. Paramyxoviridae – MeV

**Fig. 6.**
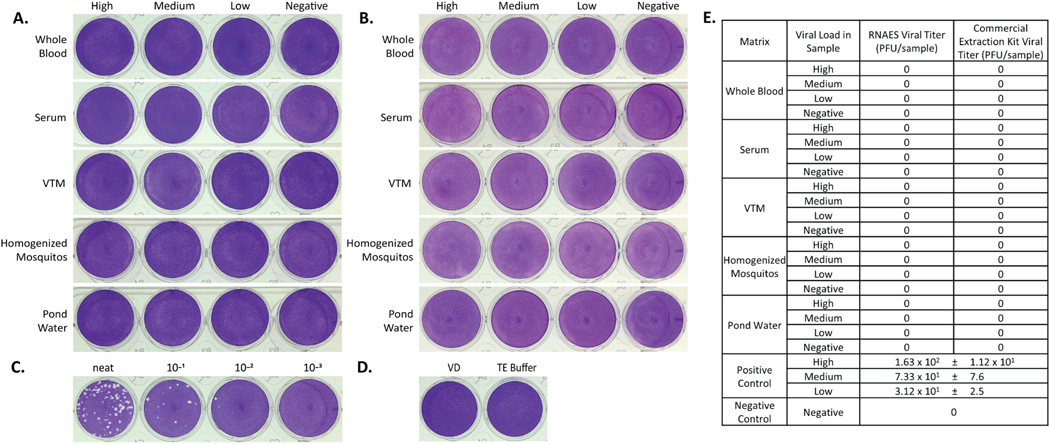
MeV is inactivated by both RNAES and Qiagen QIAamp extraction kits. MeV was spiked into five matrices with high, medium, low, and negative viral loads, followed by inactivation by two RNA extraction methods. Inactivation of eluates from the (A) RNAES and (B) Qiagen RNA extraction kits was tested using a crystal violet stained plaque assay (n = 9). (C) A positive control (n = 6) was included, the high titer positive is shown, as well as (D) a VD negative control (n = 9) and a TE buffer negative control (n = 6). (E) Summary of plaque assay results; data are expressed as mean ± SEM.

A prototype member of the *Paramyxoviridae* family, MeV, was selected to challenge the inactivation potential of the RNAES and Qiagen methods. High, medium, low, and negative viral load samples were prepared in the five matrices used for this study and each sample was tested with the RNAES extraction method, and the commercial comparator. The eluates were tested in a plaque assay, and no plaques were observed for RNAES (Fig. 6A) or the commercial comparator (Fig. 6B), suggesting that the virus in each sample was inactivated. The high viral load positive control (Fig. 6C) was determined to contain 1.63 × 10^2^ PFU in the plaque assay. The negative control, consisting of wells of TE Buffer or VD, did not have plaques (Fig. 6D). A summary of the plaque assay results for the MeV sample eluates is shown in Fig. 6E. The RNAES and Qiagen methods of nucleic acid extraction both were able to inactivate at least 1.63 × 10^2^ PFU of MeV in all tested matrices.

### 3.5. Phenuiviridae – HRTV

**Fig. 7.**
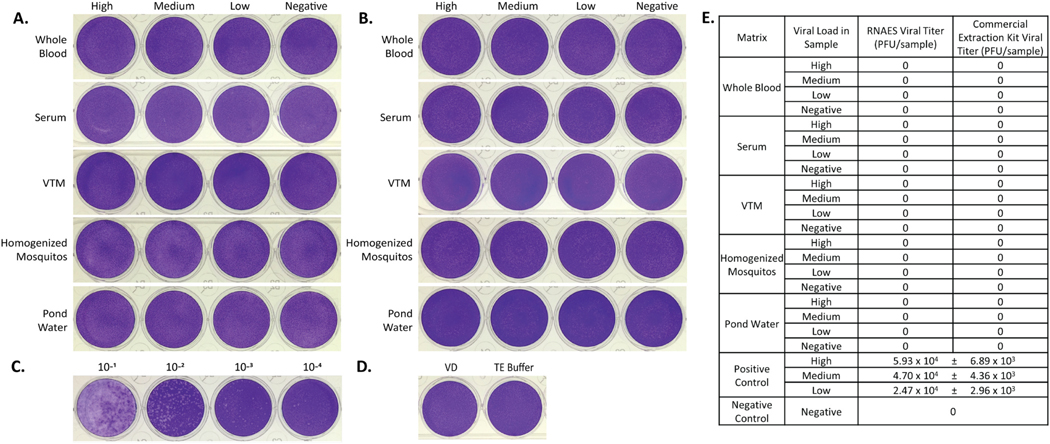
HRTV is inactivated by both RNAES and Qiagen QIAamp extraction kits. HRTV was spiked into five matrices with high, medium, low, and negative viral loads, followed by inactivation by two RNA extraction methods. Inactivation of eluates from the (A) RNAES and (B) Qiagen RNA extraction kits was tested using a crystal violet stained plaque assay (n = 9). (C) A positive control (n = 3) was included, shown is the high titer positive, as well as (D) a VD negative control (n = 6) and a TE buffer negative control (n = 3). (E) Summary of plaque assay results; titers are presented as mean ± SEM.

The prototype virus chosen to represent *Phenuiviridae* was HRTV. Samples of human whole blood, human serum, viral transport medium, homogenized mosquitos, and pond water were prepared using high, medium, low and negative viral loads. The samples were used in the RNAES RNA extraction method and Qiagen commercial comparator and eluates were tested in a plaque assay for infectious virus. No plaques were observed in any of the virus-spiked matrices purified using RNASE or commercial kit (Fig. 7A-B). The high positive control (Fig. 7C) was experimentally determined to contain 5.93 × 10^4^ PFU, indicating that the RNAES and Qiagen methods inactivate samples that contain up to 5.93 × 10^4^ PFU HRTV. The negative control contained no plaques, as predicted (Fig. 7D). A summary of all the plaque assay results is shown in Fig. 7E.

### 3.6. Togaviridae – CHIKV and WEEV

**Fig. 8.**
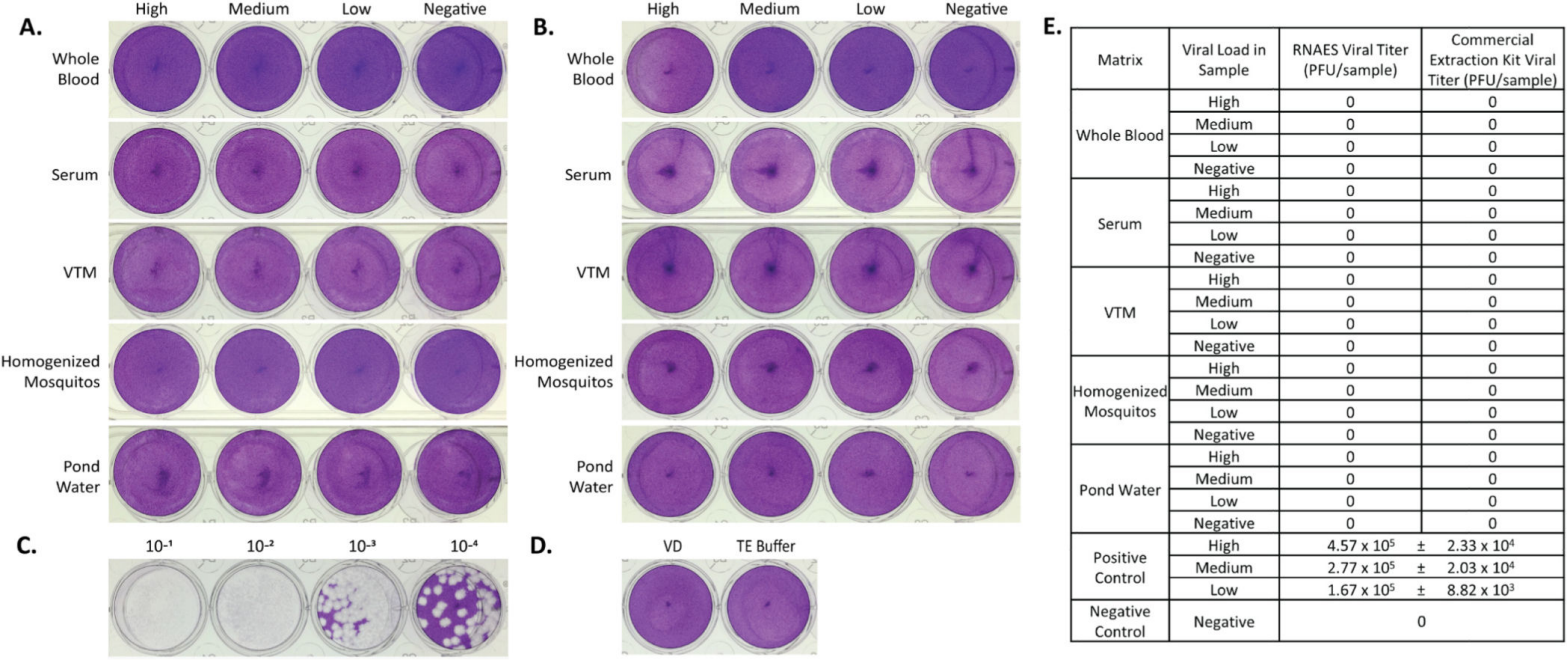
CHIKV is inactivated by both RNAES and Qiagen QIAamp extraction kits. CHIKV was spiked into five matrices with high, medium, low, and negative viral loads, followed by inactivation by two RNA extraction methods. Inactivation of eluates from the (A) RNAES and (B) Qiagen RNA extraction kits was tested using a crystal violet stained plaque assay (n = 9). (C) A high titer positive control (n = 3) was included, as well as (D) a VD negative control (n = 6) and a TE buffer negative control (n = 6). (E) Summary of plaque assay results, which are reported as mean ± SEM.

CHIKV, an old-world alphavirus, was used to spike the five tested matrices to establish high, medium, low, and negative viral load samples as a prototype *Togaviridae* family member. The RNAES method and commercial comparator kit were used to extract RNA from the virus-spiked samples, and the eluates were tested in a plaque assay. None of the eluates from the tested samples produced plaques (Fig. 8A-B). The high positive control that was 4.57 × 10^5^ PFU (Fig. 8C) in the plaque assay, suggests that both the RNAES method and commercial kit can inactivate up to 4.57 × 10^5^ PFU of CHIKV. There were also no plaques from the negative control (Fig. 8D). The plaque assay data are summarized in Fig. 8E.

**Fig. 9.**
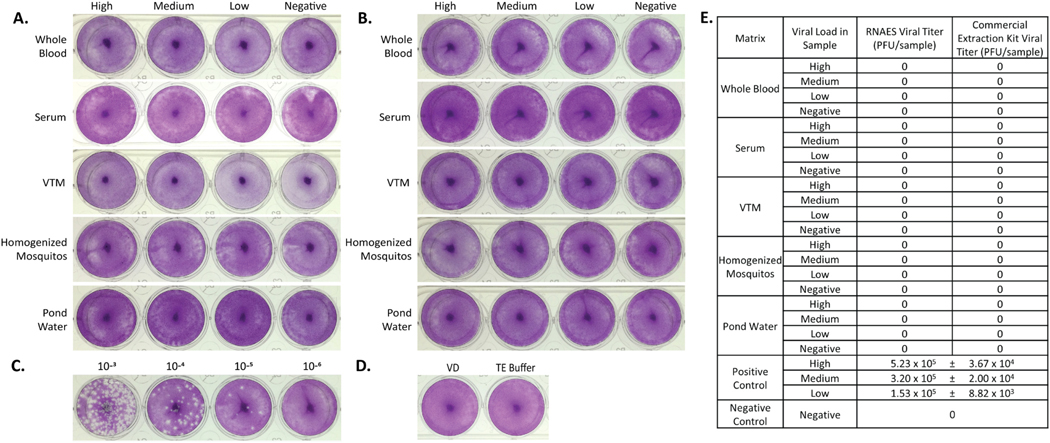
WEEV is inactivated by both RNAES and Qiagen QIAamp extraction kits. WEEV was spiked into five matrices with high, medium, low, and negative viral loads, followed by inactivation by two RNA extraction methods. Inactivation of eluates from the (A) RNAES and (B) Qiagen RNA extraction kits was tested using a crystal violet stained plaque assay (n = 9 except high viral load homogenized mosquitos for RNAES and Qiagen n = 8). (C) A positive control (n = 3) of the high titer virus was included, as well as (D) a VD negative control (n = 6) and a TE buffer negative control (n = 6). (E) Summary of plaque assay results; values are reported as mean ± SEM.

WEEV, a new-world alphavirus, was also used as a prototype *Togaviridae* virus and was used to prepare samples with high, medium, low, and zero PFU per sample. These samples were also used with the RNAES and commercial comparator extraction methods and the eluates tested in a plaque assay. There were no plaques in any of the tested conditions (Fig. 9A-B). The high viral titer positive control (Fig. 9C) had a viral load of 5.23 × 10^5^ PFU per sample in the plaque assay, suggesting that both methods can inactivate at least 5.23 × 10^5^ PFU of WEEV. There were no plaques detected in the negative control plate (Fig. 9D). Fig. 9E provides a summary of all the plaque assay data.

### 3.7. Picornaviridae – EV A71

**Fig. 10.**
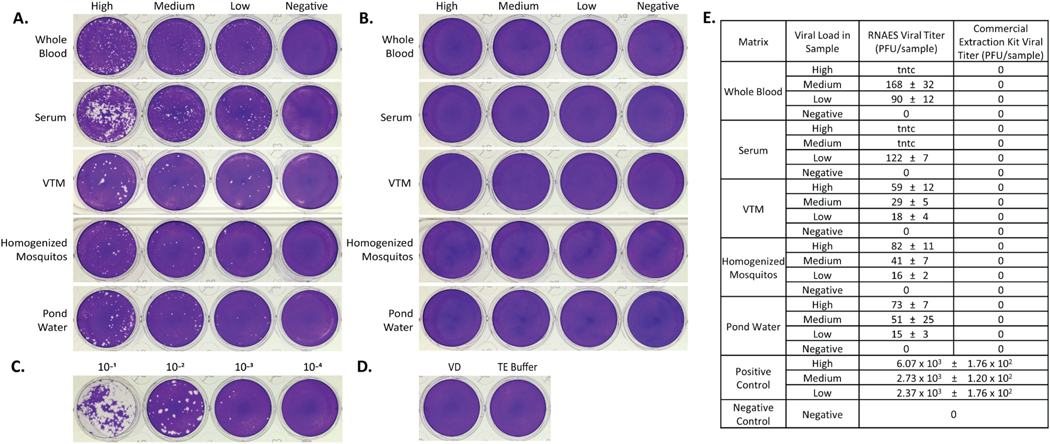
EV A71 is not completely inactivated by RNAES extraction kit. EV A71 was spiked into five matrices with high, medium, low, and negative viral loads, followed by inactivation by two RNA extraction methods. Inactivation of eluates from the (A) RNAES and (B) Qiagen RNA extraction kits was tested using a crystal violet stained plaque assay (n = 9). (C) A positive control (n = 3) was included, a representative of the high titer virus is shown, as well as (D) a VD negative control (n = 6) and a TE buffer negative control (n = 6). (E) Summary of plaque assay results, which are reported as mean ± SEM. Within the table “tntc” indicates the number of plaques was too numerous to count.

EV A71, a *Picornaviridae* family prototype virus, was spiked into the five matrices being tested at a high, medium, low, and negative concentration per sample. The spiked samples were used in both the RNAES protocol and a Qiagen commercial comparator kit. The resulting eluates were tested in a plaque assay for infectious virus. Plaques were observed in all test conditions, except the negatives (0 PFU) for the samples extracted with the RNASE method (Fig. 10A). All samples were inactivated with the Qiagen extraction kit (Fig. 10B). The high concentration positive control was determined to be 6.07 × 10^3^ PFU in the plaque assay (Fig. 10C), and no plaques were observed in the negative control plate (Fig. 10D). Although there is a reduction in bioburden of each sample with the RNASE method, the presence of plaques indicates a failure to effectively inactivate this surrogate Picornaviridae virus. A summary of the plaque assay data for EV A71 is shown in Fig. 10E.

### 3.8. RT-qPCR

Although it is important to ensure that all pathogens are inactivated, it is also essential that the RNA from the RNAES extraction kit is of high enough quality that it is usable in downstream applications. After the wash step in the RNAES workflow, membranes were transferred to storage tubes with desiccant, dried and maintained at ambient temper atures (approximately 22°C) for 19–28 days. Sample tubes were shipped by overnight courier in a standard envelope to Emory University for testing. To indirectly test the quality of the RNAES extracted RNA, viral RNA was amplified in virus specific RT-qPCR reactions. RNA was detected from all replicates prepared with all prototype viruses in all sample matrices (Table 4).

## 4. Discussion

To conduct research in challenging environments with limited resources, a method for pathogen nucleic acid extraction is necessary. The CREID Network-developed novel RNAES extraction method, which meets the required criteria, successfully inactivated surrogate/prototype viruses from six of seven virus families tested (*Coronaviridae, Flavivir idae, Hantaviridae, Paramyxoviridae, Phenuiviridae,* and *Togaviridae*) spiked into five matrices (human whole blood, human serum, viral transport medium, homogenized mosquitos, and pond water) that would be commonly collected at a field site. Additionally, RT-qPCR analysis indicated that all eluates generated through RNAES and stored at ambient temperatures (approximately 22°C) for up to a month retained RNA of sufficient quality for amplification and detection. Additionally, we have long read sequencing data and have been able to generate full-length flavivirus cDNA following RNAES extraction and sequencing of dengue and chikungunya strains on an Oxford Nanopore MinION device (unpublished). It has been shown that storage of samples in QIAamp AVL with or without ethanol impacts RNA quality (Lewandowski et al., 2017).

The Qiagen QIAamp commercial RNA extraction kit, run side-by-side with the RNAES method, was similarly able to provide non-infectious RNA samples for the same viruses, and additionally inactivated samples spiked with EV A71, from the *Picornaviridae* family.

The affordable materials for the RNAES method do not require specialized equipment (e.g., centrifuges) or a power source and make field RNA extractions more accessible. Additionally, this method allows researchers to safely ship these samples at environmental temperatures, eliminating the need for cold chain storage and shipping. The QIAamp workflow requires the full extraction and chemical waste disposal when the sample arrives in the laboratory rather than simple rehydration. Shipping corrosive and flammable chemicals requires specific precautions, and once extraction is completed, the samples again require freezer storage. This could augment the potential for surveillance capabilities, allowing earlier recognition of the presence of pathogens with pandemic potential in susceptible areas, subsequently decreasing response time.

The drawback to the RNAES method is that it was unsuccessful in complete inactivation of the only non-enveloped virus tested, EV A71. Further studies would be needed to determine whether this finding is consistent for other non-enveloped viruses using the RNAES method. Although there was an average 2.3-log reduction in bioburden of the EV A71 samples, incomplete inactivation is likely the result of the lysis chemistry not being sufficient to fully disrupt the icosahedral capsid. The RNAES was designed to disrupt viral envelopes through balancing kosmotropic and chaotropic salts in the solution. Alternatively, the commercial extraction kit, which uses guanidinium isothiocyanate, an EPA-approved germicide with upregulated waste disposal requirements, successfully inactivated the virus in all EV A71 samples. Future work to enhance the RNAES method for the inactivation of non-enveloped viruses will involve use of a binding solution containing additional chaotropes, such as urea, and low-concentration detergents to more efficiently lyse these viruses.

A limitation of the study is that we have not determined a high threshold that cannot be inactivated for six of the virus families (eight viruses). This could indicate that the kit will inactivate all virus in a sample regardless of titer or that the viral loads tested in this validation were not high enough to exceed the capabilities of the inactivation kits. This limitation could be of larger consequence when inactivating high-titer viral stock cultures, whereas for clinical samples, levels of CHIKV and WNV RNA in eluates from this study far exceed those found in patients (Gomez et al., 2015; Waggoner et al., 2016). Despite this limitation, data included in this study show the utility of the RNAES kit to safely extract nucleic acid of sufficient quality for downstream molecular assays, using prototype/surrogate viruses from six virus families, independent of sample matrix (i.e., human blood, swab, arthropod homogenate, environmental water).

The RNAES extraction method successfully inactivated eight viruses from multiple viral families that have pandemic potential. Although unsuccessful in inactivating a non-enveloped virus, the RNAES method still offers benefits in cost and ease of use making it suitable for field use, which could ultimately enhance pathogen surveillance and facilitate quicker detection of potential pandemic threats.

## Figures and Tables

**Table 1:** Virus families and prototype members used for inactivation testing.

Virus Family	Structure	Notable Members	Selected Prototype Virus	Biosafety Level (BSL)

*Coronaviridae*	Spherical, Enveloped, ssRNA (+ sense)	SARS-CoV SARS-CoV− 2 MERS-CoV	Middle Eastern Respiratory Syndrome Coronavirus (MERS-CoV)	BSL–3
*Flaviviridae*	Spherical, Enveloped, ssRNA (+ sense)	West Nile Virus Zika Virus	West Nile Virus (WNV)	BSL–2 +
		Dengue Virus Japanese Encephalitis Virus	Japanese Encephalitis Virus (JEV)	BSL–3
*Hantaviridae*	Spherical, Enveloped, Segmented RNA (- sense)	Arenaviruses Hantaviruses	Hantaan Virus (HTNV)	BSL–3
*Phenuiviridae*	Spherical, Enveloped, Segmented RNA (- sense)	Phenuiviruses	Heartland Virus (HRTV)	BSL–3
*Togaviridae*	Spherical, Enveloped, ssRNA (+ sense)	Chikungunya Virus Sindbis Virus Venezuelan Equine Encephalitis Virus	Chikungunya Virus (CHIKV)	BSL–3
		Eastern Equine Encephalitis Virus Western Equine Encephalitis Virus	Western Equine Encephalitis Virus (WEEV)	BSL–3
*Paramyxoviridae*	Pleomorphic, Enveloped, ssRNA (- sense)	Hendra Virus Nipah Virus Mumps Virus Measles Virus Human Parainfluenza Viruses	Measles Virus (MeV)	BSL–2 +
*Picornaviridae*	Icosahedral Capsid, ssRNA (+ sense)	Enterovirus A71 Echoviruses Enterovirus C Rhinovirus C Hepatovirus A	Enterovirus A71 (EV A71)	BSL–2 +

**Table 2:** Mock field sample types for inactivation testing.

Sample Type	Representative Source	Matrix	Components

Host Blood	Human	Whole blood	Innovative Research, IWB1K2E10ML
		Serum	Sigma Aldrich, H5667-mL
Host Swab	Nasopharyngeal (NP) Swab	Viral Transport Medium (VTM)	HBSS (Gibco 24020–117) + 2 % FBS + 1 % P/S + 5 % artificial saliva (Pickering 1700–0316)
Vector Pool	Homogenized Mosquitos	1X Phosphate Buffered Saline (PBS)	15–20 mosquitos/mL PBS, *Aedes aegypti* BEI MRA–735B and *Anopheles albimanus* BEI MRA–133B
Environmental	Pond water	Pond water (Durham, NC)	locally acquired

**Table 3:** Virus propagation and plaque assay conditions.

Virus Name	Propagation		Plaque Assay			
	MOI	Duration (Days)	Virus Diluent	Overlay	Duration (Days)	Stain

MERS-CoV	0.3	2	VD1	2X MEM + 0.6 % MC	2	Crystal Violet
JEV	0.001	7	VD2	2X MEM + 1 % MC	7	Crystal Violet
WNV	0.05	3	VD2	2X MEM + 1 % MC	4	Crystal Violet
HTNV	0.05	12	VD1	2X MEM + 0.6 % MC	7	Immunostain
MeV	3.5 × 10^− 6^	5	VD1	2X MEM + 0.6 % MC	5	Crystal Violet
HRTV	0.002	9	VD1	2X MEM + 0.6 % MC	9	Crystal Violet
EV A71	0.001	3	VD1	2X MEM + 0.45 % MC	4	Crystal Violet
CHIKV	0.01	2	VD1	2X MEM + 0.6 % MC	3	Crystal Violet
WEEV	0.0001	2	VD1	2X MEM + 0.6 % MC	2	Crystal Violet

**Table 4:** Viral RNA from RNAES membranes stored and shipped at ambient temperature (approximately 22°C) is of sufficient quality for amplification by RT-qPCR.

	Replicates detected/total tested (mean Ct, SD)				
Virus	Whole Blood	Serum	Homogenized mosquitos	Pond water	VTM

MERS-CoV	3/3 (19.9, 2.1)	3/3 (19.0, 1.0)	3/3 (16.2, 0.6)	3/3 (16.0, 1.0)	3/3 (15.3, 0.7)
JEV	3/3 (21.5, 0.7)	3/3 (19.6, 1.2)	3/3 (22.3, 0.8)	3/3 (18.6, 1.1)	3/3 (19.5, 1.4)
WNV	3/3 (19.6, 0.5)	3/3 (20.5, 0.5)	3/3 (18.5, 1.0)	3/3 (17.7, 1.4)	3/3 (17.8, 2.2)
HNTV	3/3 (31.0, 3.4)	3/3 (29.2, 1.1)	3/3 (24.3, 0.7)	3/3 (24.1, 0.2)	3/3 (24.1, 0.2)
MeV	3/3 (28.6, 2.1)	3/3 (27.9, 1.0)	3/3 (26.2, 0.5)	3/3 (25.7, 0.9)	3/3 (24.5, 0.5)
HRTV	3/3 (27.5, 1.6)	3/3 (26.7, 0.5)	3/3 (23.6, 0.3)	3/3 (24.3, 2.4)	3/3 (23.3, 0.4)
EV A71	3/3 (30.7, 2.8)	3/3 (28.1, 0.8)	3/3 (26.3, 0.9)	3/3 (26.6, 1.3)	3/3 (24.8, 0.9)
CHIKV	3/3 (18.9, 1.0)	3/3 (18.0, 0.6)	3/3 (15.9, 0.6)	3/3 (15.5, 0.0)	3/3 (14.0, 0.3)
WEEV	3/3 (21.8, 3.0)	3/3 (17.4, 0.9)	3/3 (15.7, 0.4)	3/3 (14.8, 1.0)	3/3 (15.4, 1.8)
**Total**	**27/27**	**27/27**	**27/27**	**27/27**	**27/27**

## Data Availability

Data will be made available on request.
